# Rapid Characterization of Undeclared Pharmaceuticals in Herbal Preparations by Ambient Ionization Mass Spectrometry for Emergency Care

**DOI:** 10.1021/jasms.4c00016

**Published:** 2024-04-15

**Authors:** Chi-Wei Lee, Hung Su, Yi-Wen Hsu, Lin-Zhen Su, Yen-Hung Wu, Chia-Yi Hou, Shu-Yu Shih, Jentaie Shiea

**Affiliations:** † Institute of Medical Science and Technology, National Sun Yat-Sen University, Kaohsiung 804201, Taiwan, ROC; ‡ Department of Chemistry, National Sun Yat-Sen University, Kaohsiung 804201, Taiwan, ROC; § Department of Emergency Medicine, Kaohsiung Medical University Hospital, Kaohsiung Medical University, Kaohsiung 80756, Taiwan, ROC; ∥ Department of Clinical Pathology, Chi-Mei Medical Center, Liouying 73659, Taiwan, ROC; ⊥ Department of Emergency Medicine, Chi-Mei Medical Center, Liouying 73659, Taiwan, ROC; # Department of Medicinal and Applied Chemistry, Kaohsiung Medical University, Kaohsiung 80756, Taiwan, ROC; ∇ Rapid Screening Research Center for Toxicology and Biomedicine, National Sun Yat-Sen University, Kaohsiung 804201, Taiwan, ROC; ○ Research Center for Environmental Medicine, Kaohsiung Medical University, Kaohsiung 80756, Taiwan, ROC

**Keywords:** acute poisoning, adulteration of herbal preparation, thermal desorption-electrospray ionization mass spectrometric platform, emergency care

## Abstract

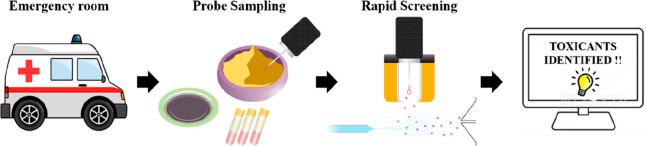

In Asia, some herbal preparations have been found to be adulterated with undeclared synthetic medicines to increase their therapeutic efficiency. Many of these adulterants were found to be toxic when overdosed and have been documented to bring about severe, even life-threatening acute poisoning events. The objective of this study is to develop a rapid and sensitive ambient ionization mass spectrometric platform to characterize the undeclared toxic adulterated ingredients in herbal preparations. Several common adulterants were spiked into different herbal preparations and human sera to simulate the clinical conditions of acute poisoning. They were then sampled with a metallic probe and analyzed by the thermal desorption-electrospray ionization mass spectrometry. The experimental parameters including sensitivity, specificity, accuracy, and turnaround time were prudently optimized in this study. Since tedious and time-consuming pretreatment of the sample is unnecessary, the toxic adulterants could be characterized within 60 s. The results can help emergency physicians to make clinical judgments and prescribe appropriate antidotes or supportive treatment in a time-sensitive manner.

## 1 Introduction

Amidst the fusion of tradition and modernity, the popularity of herbal preparations thrives. These remedies, grounded in age-old healing practices like Ayurveda and Traditional Chinese Medicine, are experiencing resurgence. Propelled by a pursuit of holistic well-being and an apparent gentler approach, they are gaining prominence alongside Western pharmaceuticals. This revival signifies a seamless integration of ancient wisdom and contemporary healthcare, providing individuals with a myriad of choices for well-being in the Asian region. Regrettably, the surreptitious inclusion of undisclosed pharmaceuticals in herbal preparations has the potential to result in cases of acute poisoning, posing a looming public health crisis. As these illicit activities flourish clandestinely, consumers confront significant risks to their well-being from contaminated products, prompting the need for immediate measures to address this precarious issue.

The term “adulteration” is defined as the act of mixing synthetic chemical substances in food or medicine without a declaration, while these chemical substances are not the expected original ingredients. Improper adulteration of pharmaceuticals into herbal preparations is illegal, and partial replacement of herbal preparations with undeclared chemicals or inferior products could be a potential health crisis, which can lead to clinical symptoms of severe acute poisoning such as seizures, disturbance of consciousness, hypoglycemia, rhabdomyolysis, renal failure, liver failure, cerebral edema, intracranial hemorrhage, coma, and even death.^1^

It has been reported that different symptoms may occur after consuming the herbal preparations with undeclared addition of inadequate dose of pharmaceuticals. For examples, (1) nonsteroidal anti-inflammatory drugs like mefenamic acid can cause upper gastrointestinal tract bleeding and hypovolemic shock;^2^ (2) pyrone, aminopyrine, and phenylbutazone can cause agranulocytosis complicated by fatal sepsis;^3^ (3) diazepam, phenytoin, and glyburide can cause consciousness disturbance due to different pharmacological mechanisms;^2,4,5^ and (4) sibutramine can cause sudden cardiac death.^6^ In addition, there are many herbal preparations that have been advertised as pure natural medicinals with the effect of improving sexual dysfunction. However, according to the US Food and Drug Administration, some of these herbal preparations contain one or more undeclared pharmaceuticals such as sildenafil or its analogues (e.g., carbodenafil), a high-dose of which might cause acute poisoning and even fatality due to their hepatotoxicity.^7,8^ It is necessary to rapidly identify the adulterants to facilitate early appropriate emergency treatment.

Traditionally, most drugs in blood are analyzed by gas chromatography mass spectrometry (GC/MS) or liquid chromatography tandem mass spectrometry (LC/MS/MS).^9−16^ Although the two techniques are sensitive and reliable, in order to avoid interferences from the sample matrices, several time-consuming and labor-intensive sample pretreatment processes such as solvent extraction, filtration, concentration, and derivatization are necessary. It usually takes hours to complete analysis by using these conventional methods, so the test reports are not expected to be useful as an instant reference for critical patients in the emergency department.

In recent years, the development of ambient ionization mass spectrometry (AIMS) has greatly shortened the time required for chemical analysis from hours to tens of seconds, because it requires minimal or no sample pretreatment.^17−24^ Previous literature indicated that wooden-tip electrospray ionization mass spectrometry (WT-ESI-MS) was developed for detection of adulterated drugs in three categories of herbal dietary supplements (HDSs), namely tranquilizer, aphrodisiac, and weight-loss products,^25^ fast-switching high-voltage porous-tip electrospray ionization mass spectrometry for rapid screening of five antirheumatic drugs in antirheumatic HDSs,^26^ and direct analysis in real time mass spectrometry (DART-MS) was performed for the characterization of seven synthetic antidiabetic drugs used as adulterants in HDSs.^27^ The thermal desorption-electrospray ionization mass spectrometry (TD-ESI/MS) is an emerging AIMS technique that can directly analyze solids, liquids, and semivolatiles. For sampling liquid, an inoculating metal probe is dipped into the sample solution to collect small amount (1–2 μL) of solution on the probe.^24^ For sampling solid, the probe loop is used to scrape gently on the surface of the sample to collect trace amount of sample on the probe surface.^24^ After sampling, the probe is transferred to a preheated chamber for thermal desorption of the analytes in the sample. The desorbed analytes are delivered by a nitrogen stream into an electrospray plume located underneath the thermal desorption chamber, where they react with the charged solvent species to form analyte ions.^24^ The time required to complete a typical TD-ESI/MS analysis is less than 60 s, so the technique has been applied in rapid detection of various toxicants in biospecimens for emergency care.^28−40^

It is not uncommon for family members of acutely poisoned patients to bring along remains or samples of suspected substances which might have led to the poisoning event to the emergency department together with the patient. If such samples could be rapidly analyzed to characterize the toxicants in them, they might end up to be the most helpful first-hand information for the emergency physicians to determine on the most appropriate antidotes or supportive care. In this study, an analytical platform based on TD-ESI/MS was developed to rapidly characterize the adulterants in herbal preparations to speed up therapeutic treatment of acutely poisoned patients in the emergency department. The parameters for charactering the adulterants with TD-ESI/MS were optimized and LC/MS/MS was used to validate the results. It is expected that the developed analytical platform can solve the bottleneck of the traditional methods which help the emergency physicians to make the most correct clinical judgments and treatments.

## 2 Materials and Methods

### 2.1 Chemicals, Materials, and Sample Preparations

Standards of adulterants commonly found in herbal preparations including acetaminophen, caffeine, mefenamic acid, phenytoin, sibutramine, diazepam, phenylbutazone, and sildenafil were purchased from ChemScene (Monmouth Junction, NJ, U.S.A.). HPLC-grade methanol (MeOH) and acetone were purchased from Merck (Darmstadt, Germany), while hexane and ethyl acetate (EA) were purchased from J.T. Baker (Phillipsburg, NJ, U.S.A.). Acetic acid was obtained from Sigma-Aldrich (St. Louis, MO, U.S.A.). Distilled deionized water (purified with a PURELAB Classic UV from ELGA, Marlow, U.K.) was used to prepare the electrospray solution (50% methanol containing 0.1% acetic acid, v*/*v). All chemical reagents and solvents were used without additional purification.

The powders of herbal preparations including Shi Shen Tang, Mu Fang Ji Tang, Long Dan Xie Gan Tang, and Shih Chuan Tu Pu Tang were purchased from local traditional medicine stores. Homemade #1 and #2 were prepared according to the homemade recipe. Table 1 shows the ingredients of these herbal preparations. All samples were stored at 4 °C prior to the analysis. Human serum was purchased from the Innovative Research Incorporation (Oakland, CA, U.S.A.). The biospecimens were stored at −80 °C for subsequent analysis.

**Table 1:** Ingredients of the Herbal Preparations Used in This Study

herbal preparations	ingredients
Shi Shen Tang	*Citri Reticulatae Pericarpium*, *Ephedra sinica*, *Chuanxiong Rhizome*, *Glycyrrhiza uralensis* (roasted radix root), *Cyperus rotundus*, *Perillae Folium*, *Angelica dahurica*, *Actaea cimicifuga*, *Radix Puerariae*, *Paeoniae Radix Rubra*, *Zingiberis Rhizoma*, starch
Long Dan Xie Gan Tang	*Gentiana scabra*, *Scutellaria baicalensis*, *Gardenia jasminoides*, *Alisma plantago-aquatica*, *Akebiae Caulis*, *Plantago psyllium*, *Angelica sinensis*, *Rehmannia glutinosa* (fresh rehmannia root), *Bupleuri Radix*, *Glycyrrhiza uralensis*, starch, fiber
Snailseed soup	*Stephania tetrandra*, midicinal plaster, *Ramulus Cinnamomi*, *Panax ginseng*
Shih Chuan Tu Pu Tang	*Poria Cocos*, *Atractylodes macrocephala*, *Panax ginseng*, *Rehmannia glutinosa* (processed rehmannia root), *Paeoniae Alba*, *Glycyrrhiza uralensis* (roasted radix root), *Astragalus membranaceus*, *Cinnamomum cassi*, *Angelica sinensis*, *Chuanxiong Rhizome*, *Zingiberis Rhizoma*, *Jujubae Fructus*, starch
Homemade #1	*Semen Cassiae*, *Alisma plantago-aquatica*, *Scutellaria baicalensis*, *Glycyrrhiza uralensis*
Homemade #2	*Scutellaria baicalensis*, *Panax ginseng*, *Angelica sinensis*, *Chuanxiong Rhizome*

For TD-ESI/MS/MS analysis, the powder sample was prepared by adding 100 μL of the methanol solution containing 100 μg/mL of adulterant into 1 g of herbal powder. The powder sample was vortexed for 1 min to ensure homogeneous. The decoction was prepared by vortex 100 mg herbal powder with 10 mL pure water for 1 min. The decoction was then spiked with adulterant and 2 μL of the solution was collected by a micropipette for analysis. To simulate an actual clinical situation of acute poisoning, simple solvent extraction was performed as follows: the 900 μL human serum was spiked with 100 μL adulterant first, then 200 μL of the mixture was extracted with 400 μL EA by gentle votex for 20 s. The mixture was centrifuged for 2 min at 6000 rpm, and the supernatant (organic phase) was transferred to a glass vial for subsequent direct analysis. The turnaround time took approximately less than 5 min.

The results obtained from the analysis done with TD-ESI/MS/MS were validated using the LC/MS/MS approach. The 100 mg herbal powder, 1 mL herbal decoction, and 100 μL human serum were extracted with 10 mL methanol by gentle votex for 20 s. The mixture was centrifuged for 5 min at 6000 rpm, and the supernatant (organic phase) was filtrated with a 0.22 μm PVDF syringe filter (Agela Technologies, CA, U.S.A.) before transferring to a glass vial. All chromatographic parameters were well-conditioned for further LC/MS/MS analysis. The turnaround time took approximately at least 60 min.

### 2.2 Thermal Desorption-Electrospray Ionization Tandem Mass Spectrometry (TD-ESI/MS/MS) Analysis

**Figure 1:**
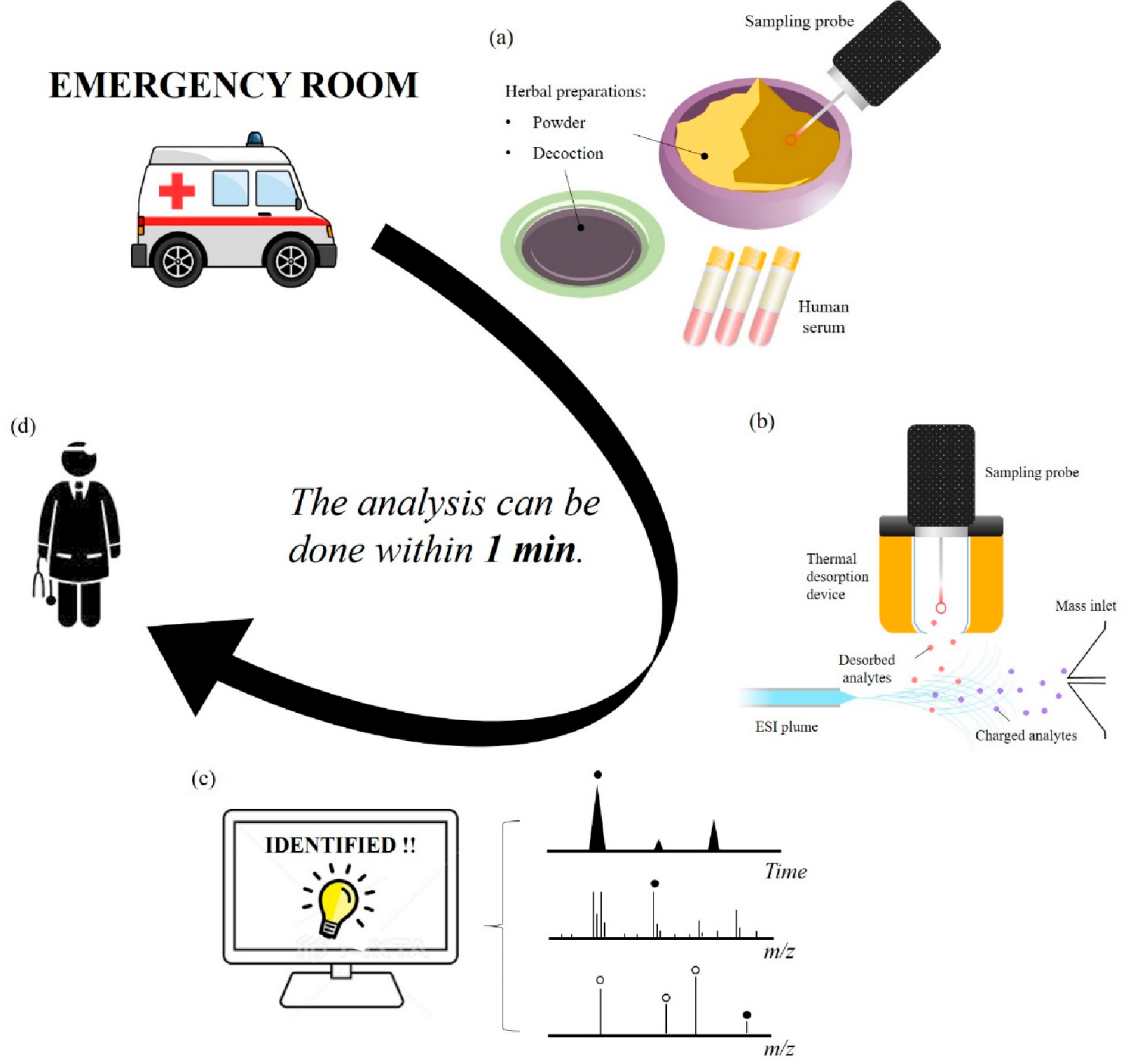
A conceptual diagram illustrating the application of TD-ESI/MS for rapid analysis of the adulterants in herbal preparations: (a) probe sampling of herbal preparations or human serum, (b) desorption of analytes from the sampling probe via thermal desorption; ionization of the desorbed analytes via their interactions with charged solvent species in an electrospray plume; and detection of analyte ions with a linear ion trap mass analyzer, (c) identification of the analyte through database library matching, and (d) the toxicological information was sent to emergency physician to determine proper therapeutic treatment.

The TD-ESI/MS system was set up in the similar design as it was described in our previous publication.^24^ The TD-ESI system is comprised of a thermal desorption unit, and an electrospray ionization device. After sampling, the probe was inserted into the TD-ESI source. The desorption temperature of the source was 300 °C using a temperature controller (ANLY AT-502, Taipei, Taiwan) and thermocouple attached to the inside of the TD-ESI unit. Nitrogen gas was preheated by passing it through in a hot metal coil (id 0.6 mm, od 1.5 mm, length 45 mm) before entering the desorption area. The hot nitrogen stream flowed (at a pressure of 5 L/min) from the top of the TD unit to deliver the desorbed analytes into the ESI plume located right below (8 mm) the exit of the TD unit. The ESI solution was comprised of MeOH/H_2_O (1:1, v*/*v) with 0.1% acetic acid (v*/*v). A high voltage (5 kV for positive ion mode and −5 kV for negative ion mode) was applied to the ESI capillary via solution conduction to induce electrospray from the solution flowing out of the capillary. The ions generated in the TD-ESI source were detected by a linear ion trap mass analyzer (LTQ XL, Thermo Fisher Scientific, Waltham, MA, U.S.A.) for MS and MS/MS analyses in positive ion mode. Qualitative determination of adulterants was based on the detection of characteristic analyte ion pairs in multiple reactions monitoring (MRM) mode. Two precursor-product ion transitions were monitored for each adulterant to ensure highly accurate identification. After each sample analysis, the sampling probe loop was cleaned by burning it with a high-temperature flame from a hand-held butane torch for 3–4 s. The probe was then dip in methanol to remove any residual organic compound. The analytical time required for probe sampling, thermal desorption, electrospray ionization, ion detection, and probe cleaning was less than 1 min. The operational processes of using TD-ESI/MS technique to detect adulterants in herbal preparations from three different types of samples were shown in Figure 1.

### 2.3 Liquid Chromatography Tandem Mass Spectrometry (LC/MS/MS) Analysis

LC/MS/MS analyses were conducted using a Shimadzu LC/MS system equipped with a binary pump (Nexera X2, LC-3AD), degasser (DGU-20A5R), autosampler (Nexera X2, SIL-30AC), column oven (CTO-20AC), and a triple quadrupole mass analyzer (LCMS-8045). A Phenyl Group C18 Column (Shim-pack GIST, 2 μm, 2.1 mm × 100 mm, Shimadzu, Japan) was used to separate different polar analytes under 40 °C. The mobile phase (A) consisted of 50 mM ammonium acetate in deionized water, while the mobile phase (B) consisted of pure methanol. During HPLC analysis, the injection volume and flow rate of mobile phase was set at 1 μL and 0.3 mL/min, and the eluted gradient of the organic mobile phase (B) was 0.0–1.0 min: 60%, 1.0–10.0 min: 60–100%, 10.0–15.0 min: 100%, 15.0–16.5 min: 100–60%, and 16.5–23.0 min: 60%. The operational parameters for the mass spectrometer were set at an interface voltage of 4.0 kV, nebulizer gas flow rate of 3 L/min, heating gas flow rate of 10 L/min, interface temperature of 300 °C, DL temperature of 250 °C, drying gas flow rate of 10 L/min, and heat block temperature of 400 °C.

## 3 Results and Discussion

An analytical platform using TD-ESI/MS to characterize adulterants in herbal preparations was developed in this study (Figure 1). The typical analytical processes involved in this platform include: (1) probe sampling of herbal preparations or human serum, (2) desorption of analytes via thermal desorption, (3) ionization of the desorbed analytes via their interactions with charged solvent species in the electrospray plume, (4) detection of the analyte ions with a linear ion trap mass analyzer, and (5) removal of residual sample on the probe by burning it with a high-temperature flame.

To evaluate the capabilities of TD-ESI/MS on the detection of adulterants in herbal preparations, eight common adulterant standards were examined to establish a relevant database for further real samples mapping. The mass spectra of the adulterant standard solutions (10 μg/mL for each) were recorded with probe sampling followed by TD-ESI/MS analysis. As shown in Figure S1a of the Supporting Information, the molecular ion (MH^+^) of all standards were detected including acetaminophen (*m*/*z* 152), caffeine (*m*/*z* 195), mefenamic acid (*m*/*z* 242), phenytoin (*m*/*z* 253), sibutramine (*m*/*z* 280), diazepam (*m*/*z* 285), phenylbutazone (*m*/*z* 309), and sildenafil (*m*/*z* 475). TD-ESI/MS/MS was also used to obtain the product ion information on each adulterant standard (Figure, S1b–i). The precursor ion and prominent product ions of each adulterant recorded by TD-ESI/MS/MS were summarized in Table 2. After the experimental parameters for analysis of the adulterant standards with TD-ESI/MS were optimized, they were used for characterizing the targeted compounds spiked in different herbal preparations. The optimized experimental parameters used in the TD-ESI/MS platform were listed in Table 3.

**Table 2:** Undeclared Adulterants Commonly Found in Herbal Preparations

herbal preparation	therapeutic effect	adulterant	formula	precursor ion (*m*/*z*)	product ion (*m*/*z*)	reference
Shi Shen Tang	antipyretic	acetaminophen	C_8_H_9_NO_2_	152, MH^+^	110*, 134	(41)
	psycho-stimulant	caffeine	C_8_H_10_N_4_O_2_	195, MH^+^	138*, 110	(42)
Mu Fang Ji Tang	analgesic	mefenamic acid	C_15_H_15_NO_2_	242, MH^+^	224*, 214	(43)
Long Dan Xie Gan Tang	analgesic	phenylbutazone	C_19_H_20_N_2_O_2_	309, MH^+^	211*, 188	(44)
Shih Chuan Tu Pu Tang	PDE5 inhibitor	sildenafil	C_22_H_30_N_6_O_4_S	475, MH^+^	377*, 311	(45)
Homemade #1	antiobesity agent	sibutramine	C_17_H_26_ClN	280, MH^+^	139*, 153	(46)
Homemade #2	anticonvulsant	phenytoin	C_15_H_12_N_2_O_2_	253, MH^+^	225*, 182	(47)
	anticonvulsant	diazepam	C_16_H_13_ClN_2_O	285, MH^+^	257*, 154	(48)

**Table 3:** Optimized Parameters for TD-ESI System

parameters	optimized setting
desorption temperature	280 °C
carrier gas (heated N_2_ stream) flow rate	5 L/min
ESI high voltage	5 kV
ESI solution flow rate	2.17 μL/min
ESI solution composition	40% MeOH + 1% FA
sheath gas (nebulizer gas) flow rate	4 L/min
distance between quartz tube and ESI capillary	8 mm
distance between ESI capillary and MS inlet	5 mm

To avoid the interferences from herbal matrices, tedious and time-consuming sample pretreatment is necessary before GC/MS and LC/MS/MS analyses. However, as most of the herbal matrices are nonvolatile, low or non-polar, they are either undesorbed or un-ionized during TD-ESI processes. This would minimize the interferences from the herbal matrices during TD-ESI/MS analysis, making direct characterization of adulterants in herbal preparation without sample pretreatment possible. The adulterants (10 μg/mL for each) spiked in exclusive herbal powder were studied with TD-ESI/MS. To efficiently collect herbal powders on the sampling probe, the probe surface was wetted by dip-and-remove from pure water; it was then inserted into the herbal powders for sampling.

**Figure 2:**
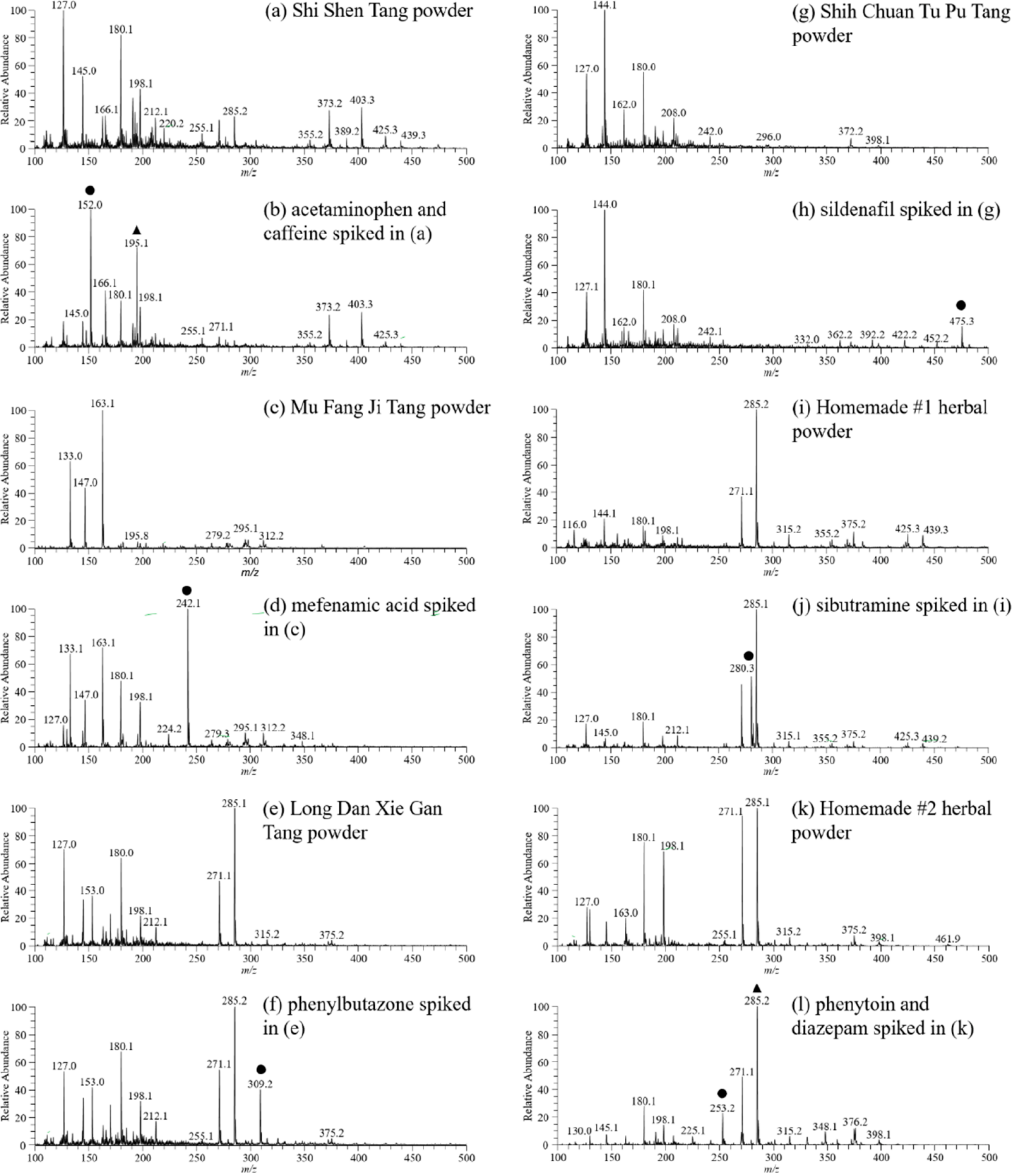
TD-ESI mass spectra obtained from (a) Shi Shen Tang powder, (b) Shi Shen Tang powder laced with acetaminophen and caffeine, (c) Mu Fang Ji Tang powder, (d) Mu Fang Ji Tang powder laced with mefenamic acid, (e) Long Dan Xie Gan Tang powder, (f) Long Dan Xie Gan Tang powder laced with phenylbutazone, (g) Shih Chuan Tu Pu Tang powder, (h) Shih Chuan Tu Pu Tang powder laced with sildenafil, (i) Homemade #1 herbal powder, (j) Homemade #1 herbal powder laced with sibutramine, (k) Homemade #2 herbal powder, and (l) Homemade #2 herbal powder laced with phenytoin and diazepam. Ten μg/mL of each standard was added into the herbal preparation powder.

Figure 2a,c,e,g,i,k shows the mass spectra recorded from six herbal powders without adulterant. The results indicated that the predominant ion signals from herbal matrix were different among the samples. The Shi Shen Tang has claimed to have analgesic, antipyretic, and psycho-stimulant effects, therefore it is often adulterated with both of acetaminophen and caffeine. Even glucose (M^+●^, *m*/*z* 180 and [M+H_2_O]^+●^, *m*/*z* 198) were detected as the predominant ion signals on the TD-ESI mass spectrum (Figure 2a), the protonated acetaminophen (*m*/*z* 152) and caffeine (*m*/*z* 195) ions were still detected (Figure 2b). The Mu Fang Ji Tang and Long Dan Xie Gan Tang were claimed to have anti-inflammatory, analgesic, and antipyretic effects, thus, they are often adulterated with NSAIDs such as mefenamic acid or phenylbutazone. However, excessive doses of NSAID could cause severe upper gastrointestinal tract bleeding and hypovolemic shock.^2,3^ Sildenafil was a phosphodiesterase 5 inhibitor (PDE5I) for the treatment of patients with erectile dysfunction,^7,8^ and it was often adulterated with the Shih Chuan Tu Pu Tang to speed up the aphrodisiac effects. Herein, TD-ESI/MS was used to detect successfully the presence of these adulterants (10 μg/mL each) spiked in the herbal powders (Figure 2b,d,f,h,j,l). It has been reported that the contents of undeclared adulterants laced in herbal powder samples usually range from 1 to 10% (w/w), which is sufficiently high to be detected by TD-ESI/MS.

It is worth noting that many homemade recipes are popular especially in the region of Asia, where the herbal preparations are claimed to have the effects of losing weight, relaxing and unwinding. Therefore, two in-house formula powders (Homemade #1 and #2) were prepared and respectively spiked with sibutramine, phenytoin, and diazepam for study. Again the three adulterants spiked in two homemade herbal powders were successfully detected with TD-ESI/MS (Figure 2j and l).

**Figure 3:**
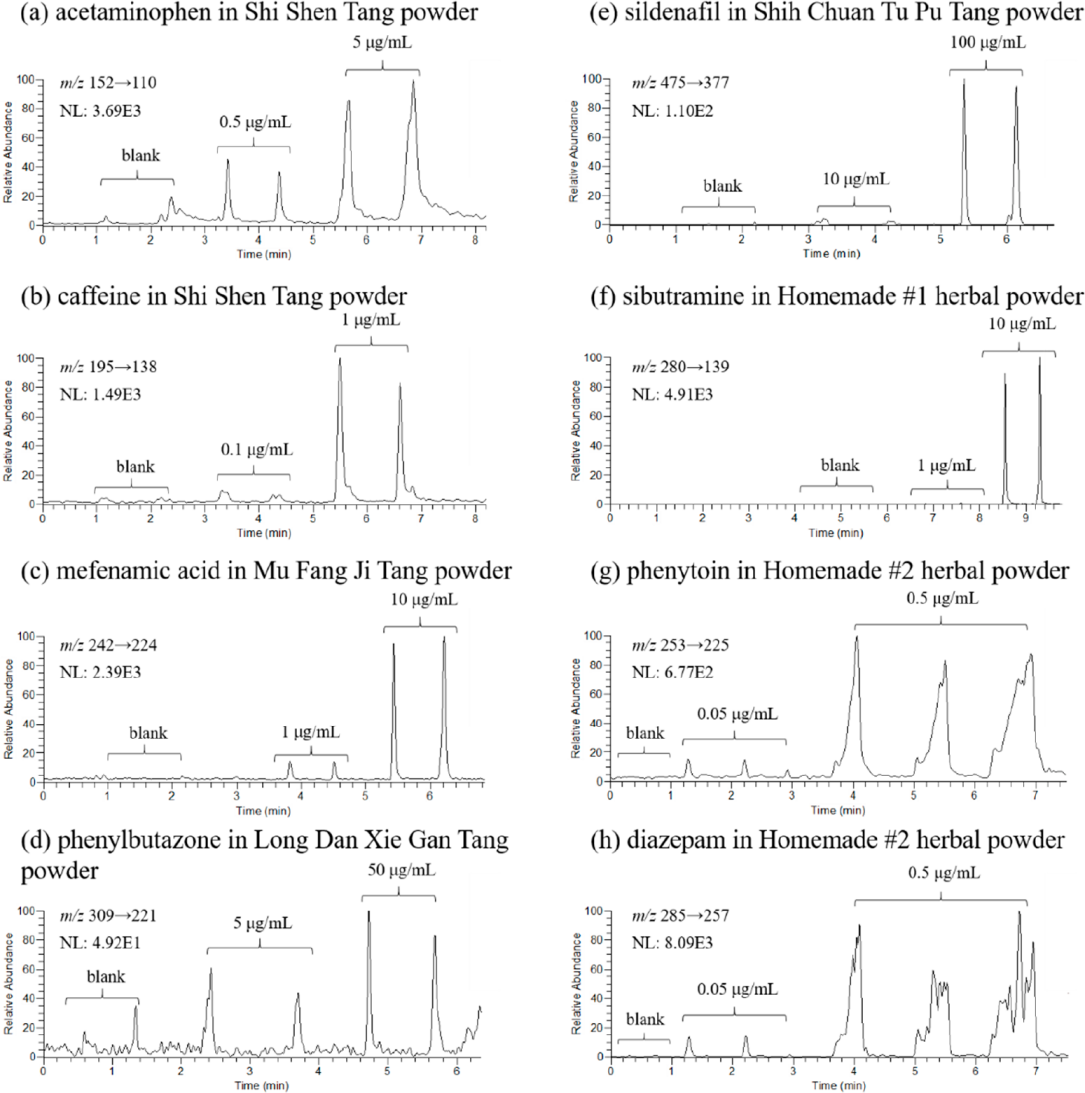
Duplicated or triplicated analytical results for using TD-ESI/MS/MS to detect the adulterant spiked in the herbal powder: (a) acetaminophen in Shi Shen Tang powder, (b) caffeine in Shi Shen Tang powder, (c) mefenamic acid in Mu Fang Ji Tang powder, (d) phenylbutazone in Long Dan Xie Gan Tang powder, (e) sildenafil in Shih Chuan Tu Pu Tang powder, (f) sibutramine in Homemade #1 herbal powder, (g) phenytoin in Homemade #2 herbal powder, and (h) diazepam in Homemade #2 herbal powder. The concentrations of the spiked adulterant are labeled in each part.

Since the sensitivity of tandem mass spectrometry (operated under MRM mode) is better than that of the mass spectrometry operated under full scan mode, TD-ESI/MS/MS was subsequently used to detect trace adulterants in the herbal preparations. Figure 3 shows the experimental results by using TD-ESI/MS/MS to analyze the herbal powder samples containing none and two different concentrations of adulterants. In addition, the messy MRMs from phenytoin and diazepam at 0.5 μg/mL were observed. The main reason for the incomplete peak shape of the extract ion current is primarily since the substrates added by the two drugs (Homemade #2) are more complex compared to others, and their composition cannot be ground into fine powder like other substrates. The results indicated that lower detection limits were achieved by TD-ESI/MS/MS than those of TD-ESI/MS for the detection of adulterant standards prepared in methanol and of those spiked in different herbal preparations (Table 4). Human serum >20 μg/mL acetaminophen,^49^ >15 μg/mL caffeine,^50^ > 1.5 μg/mL diazepam,^51^ > 20 μg/mL phenytoin,^52^ and >45 μg/mL phenylbutazone.^53^ Such overdose levels encountered in actual clinical practice are far higher than the LODs of TD-ESI/MS described in this study.

**Table 4:** Estimated Detection Limits for Detection of the Adulterants Prepared in Methanol and Spiked in Herbal Preparations by Using TD-ESI/MS/MS

	detection limit (μg/mL)		
analyte	methanol solution	herbal powder	herbal decoction
acetaminophen	0.05	0.5	0.05
caffeine	0.05	0.1	0.05
mefenamic acid	0.01	1	1
phenylbutazone	1	5	5
sildenafil	1	10	10
sibutramine	0.5	1	1
phenytoin	0.01	0.05	0.01
diazepam	0.01	0.05	0.01

Herbal decoction is made by boiling or simmering the herb powders in water, extracting its beneficial compounds to form a liquid. This method results in a potent solution often used as a drink or in various external applications due to its concentrated nature. However, the powder of an herb involves grinding the dry herb into a fine substance. This powdered form allows for easier storage, dosage measurement, and various applications including direct consumption, encapsulation for supplements, or culinary uses. While both methods aim to extract the beneficial properties of herbs, their resulting forms and applications differ significantly, with decoctions yielding a concentrated liquid and powdered herbs providing a versatile dry substance.

**Figure 4:**
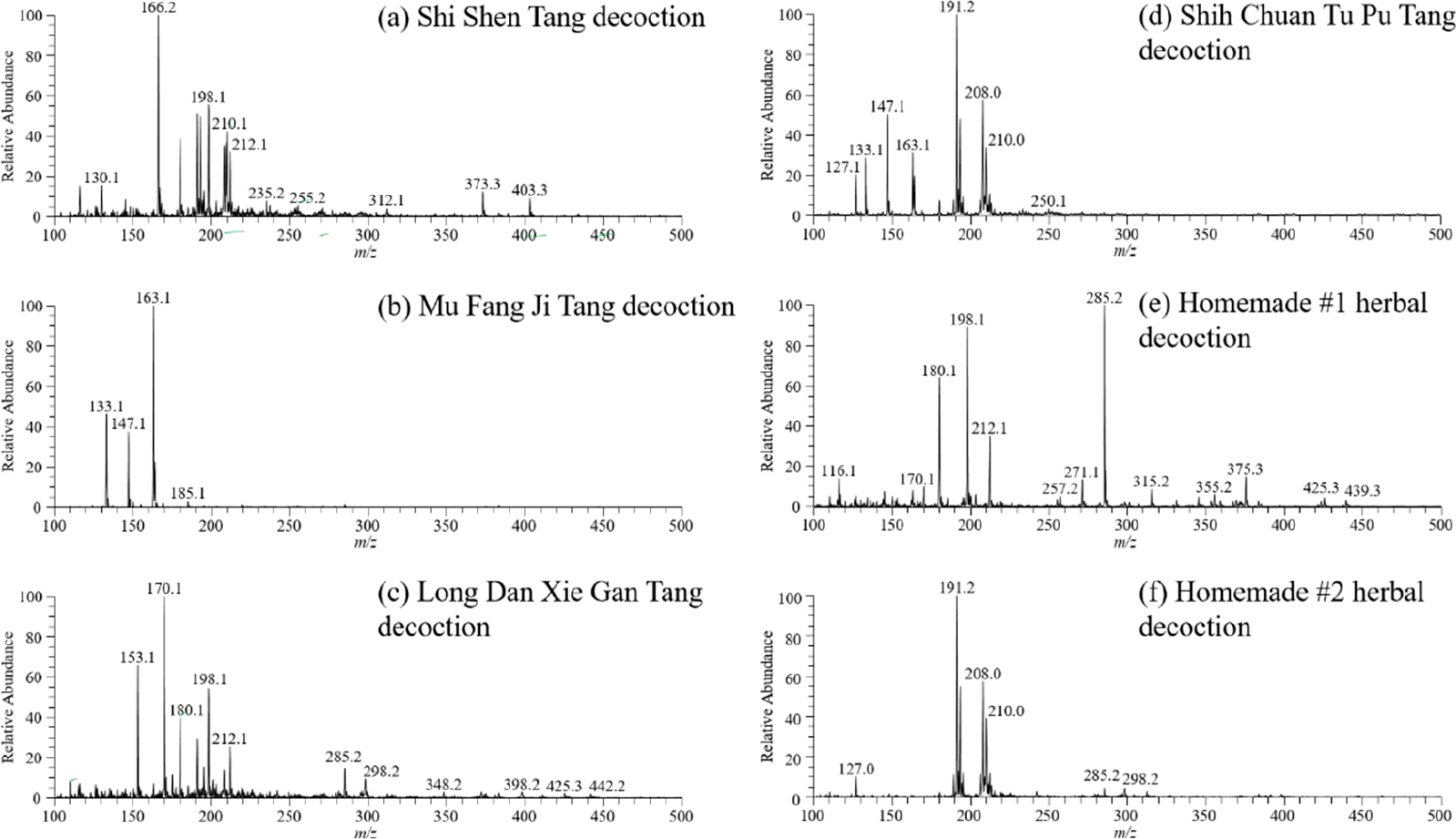
TD-ESI mass spectra of six herbal decoctions: (a) Shi Shen Tang, (b) Mu Fang Ji Tang, (c) Long Dan Xie Gan Tang, (d) Shih Chuan Tu Pu Tang, (e) Homemade #1 herbal decoction, and (f) Homemade #2 herbal decoction.

TD-ESI/MS was utilized to study chemical composition of herbal decoction. The TD-ESI mass spectra of Shi Shen Tang, Mu Fang Ji Tang, Long Dan Xie Gan Tang, and Shih Chuan Tu Pu Tang were recorded and shown in Figure 4. The TD-ESI mass spectra of Shi Shen Tang and Shih Chuan Tu Pu Tang were dominated by the ligustilide ion (*m*/*z* 191) originated from Tangkuei present in both herbal decoctions (Figure 4a and d).^54^ For Mu Fang Ji Tang, the ion of cinnamaldehyde (*m*/*z* 133) from GUI Zhi was detected (Figure 4b).^55,56^ For Long Dan Xie Gan Tang, its TD-ESI mass spectrum was dominated by the ions from spicatic acid (*m*/*z* 198) and scutellarin (*m*/*z* 285) which were origins from Huangqin (Figure 4c).^57^ To simulate the undeclared adulterant in real world situations, homemade herbal decoction #1 and #2 were also subjected for analysis and the results are shown in Figure 4e and f.

**Figure 5:**
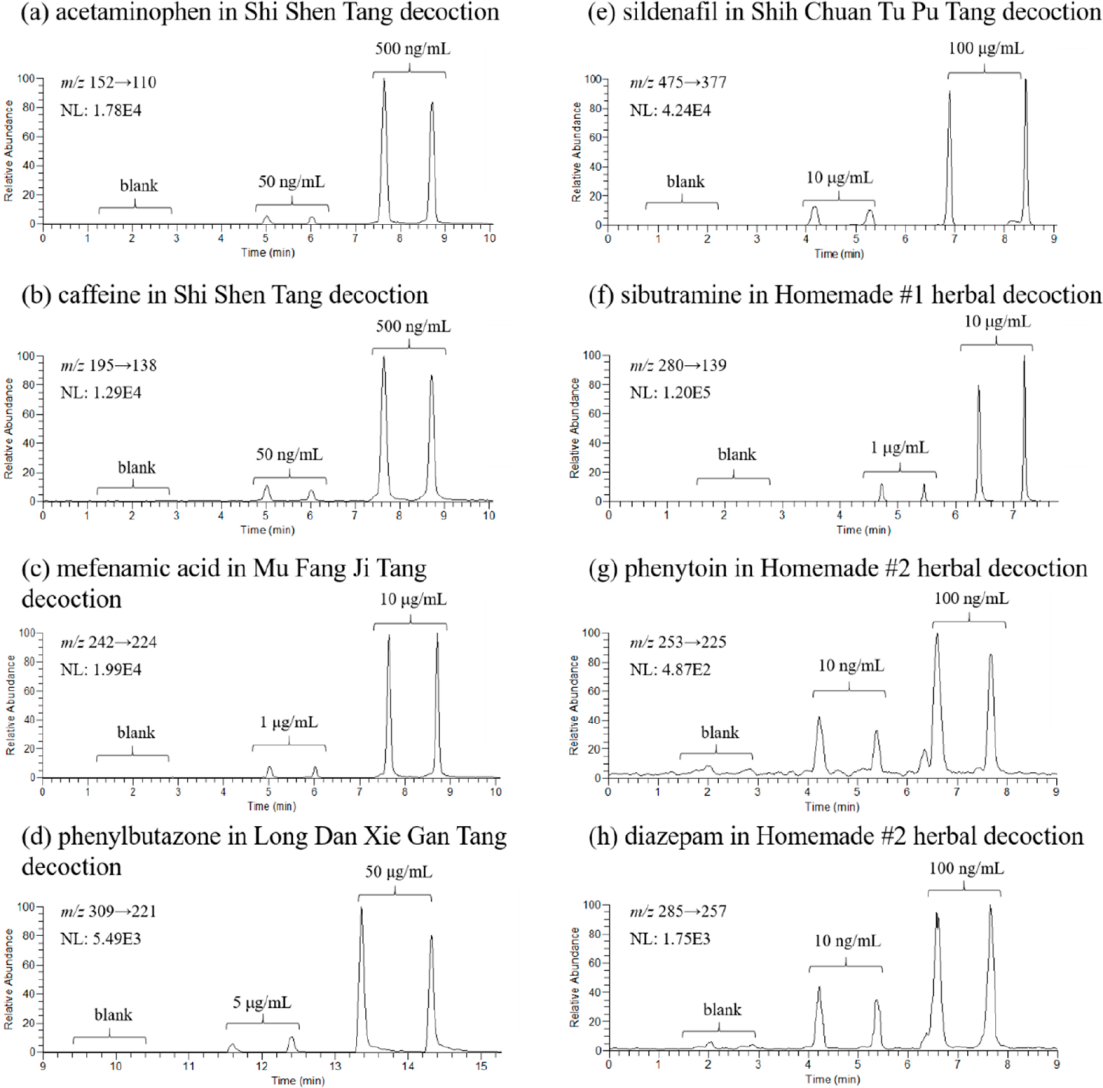
Duplicated results for using TD-ESI/MS/MS to detect the adulterant spiked in the herbal decoction: (a) acetaminophen in Shi Shen Tang decoction, (b) caffeine in Shi Shen Tang decoction, (c) mefenamic acid in Mu Fang Ji Tang decoction, (d) phenylbutazone in Long Dan Xie Gan Tang decoction, (e) sildenafil in Shih Chuan Tu Pu Tang decoction, (f) sibutramine in Homemade #1 herbal decoction, (g) phenytoin in Homemade #2 herbal decoction, and (h) diazepam in Homemade #2 herbal decoction. The concentrations of the spiked adulterant are labeled in each part.

The adulterant of different concentrations were spiked in the herbal decoctions to study the capability of using TD-ESI/MS/MS to detect the adulterants in decoction without sample pretreatment. The detection limit for acetaminophen and caffeine (spiked in Shi Shen Tang decoction), mefenamic acid (spiked in Mu Fang Ji Tang decoction), phenylbutazone (spiked in Long Dan Xie Gan Tang decoction), sildenafil (spiked in Shih Chuan Tu Pu Tang decoction), sibutramine (spiked in Homemade #1 herbal decoction), and phenytoin and diazepam (spiked in Homemade #2 herbal decoction) were found to be between 10 ng/mL (phenytoin and diazepam) and 10 μg/mL (sildenafil) in the herbal decoctions (Figure 5). Comparing the results presented in Figures 3 and 5, the detection limits for adulterants in herbal powders with TD-ESI/MS/MS was similar or higher than those in its respective decoctions, indicating that more matrix effects were found in the herbal powders (Table 3). The detection of adulterants in herbal preparations by TD-ESI/MS/MS was validated by simple solvent extraction and concentration followed by LC/MS/MS. The results are shown in Figures S2 and S3.

In toxicology, if toxic substances are only found in oral fluids, gastric lavage drainage fluid, or leftovers, they are not fully eligible to constitute the basis for the diagnosis of poisoning. Only when the toxic substances or their metabolites are detected in the victims’ blood or other body fluids should it be considered as poisoning. Since the lumen of the alimentary canal is regarded as the “external compartment” to the human body (as opposed to blood or other tissues as the “internal compartment” to the human body) in the medical point-of-view, when toxic substances are found only in the gastrointestinal lumen before being absorbed such a condition ought not be considered as a “poisoning” event. However, because human blood is rich in various biochemical compounds such as peptides, proteins, and carbohydrates. These will cause serious matrix effect during mass spectrometric analysis. This might offsets the advantages of AIMS that can rapidly identify the undeclared pharmaceutical ingredients in herbal preparations consumed and absorbed into bloodstream. Herein, we have endeavored to establish an analytical platform based on TD-ESI/MS technique to detect the adulterants in human serum for emergency care.

**Figure 6:**
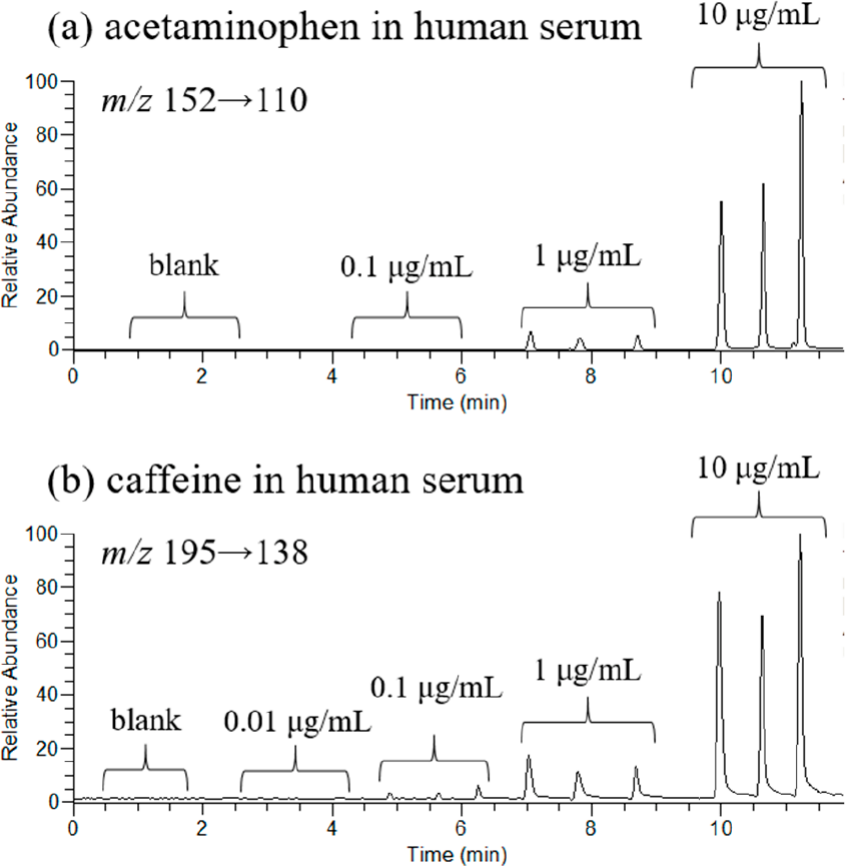
Triplicated analysis of human serum spiked with different concentrations of (a) acetaminophen (0.1, 1, and 10 μg/mL) and (b) caffeine (0.01, 0.1, 1, and 10 μg/mL) by using solvent extraction followed by TD-ESI/MS/MS analysis.

To examine the capability of TD-ESI/MS/MS to be used to detect toxins for emergency care, human serum spiked with adulterant standards was used as the sample to mimic poisoned situation. Ethyl acetate was used to mix thoroughly with the serum to rapidly extract the adulterant-containing serum. After sample pretreatment, a small amount of EA (2 μL) was withdrawn and used for TD-ESI/MS/MS analysis. The detection limit of acetaminophen in human serum by TD-ESI/MS/MS was estimated through the results for analyzing the samples spiked with three different concentrations (0, 0.1, 1, and 10 μg/mL). The detection limits of acetaminophen and caffeine were estimated to be between 0.1–1 μg/mL and 0.01–0.1 μg/mL (as shown in Figure 6a and b), respectively. This concentration should be sufficient for the developed analytical platform to be applied for real serum analysis for the poisoned patient in emergency room that usually contains higher adulterant concentration. The detection of acetaminophen in human serum by TD-ESI/MS/MS was validated by simple solvent extraction and concentration followed by LC/MS/MS (Figure S4).

**Figure 7:**
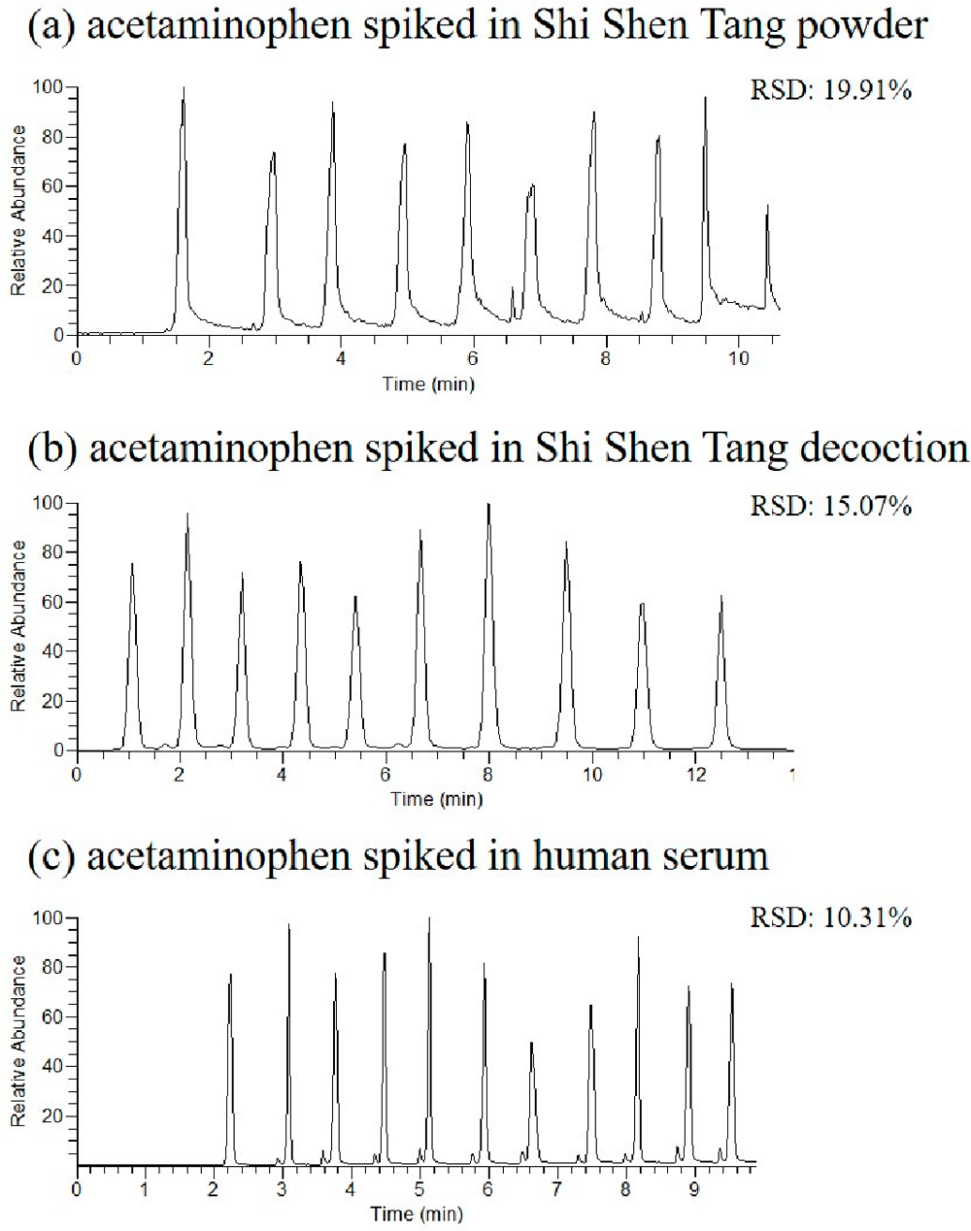
Repeatability tests (*n* = 10) for using TD-ESI/MS/MS to detect acetaminophen (10 μg/mL) spiked in different herbal matrices: (a) Shi Shen Tang powder, (b) Shi Shen Tang decoction, and (c) human serum. *The value of%RSD was calculated with the peak area of the ion signal.

The repeatability of TD-ESI/MS/MS was examined by monitoring consecutive analysis of three different sample matrices including herbal powder, herbal decoction, and human serum spiked with acetaminophen (10 μg/mL each). Figure 7 shows that the relative standard deviations (RSDs) for the detection of acetaminophen in these matrices were 19.91% (powder), 15.07% (decoction), and 10.31% (serum), respectively. The results indicated that higher RSDs were obtained from herbal powder and herbal decoction than that of human serum. The better RSD obtained from the acetaminophen spiked in human serum was found due to the sample being extracted by organic solvent prior to analysis. Compared with direct sampling, the higher RSDs originated not only from the problem of uneven mixing but also from the slightly different amounts of sample collected each time due to the manual manipulation by means of a sampling probe. However, the RSD obtained by the ambient ionization mass spectrometric platform was still technically speaking acceptable, and such a minor drawback is well offset by the high efficiency of this novel technique.

In emergency care, sporadic cases of acute poisoning are the most common scenarios, and rapid qualitative toxicant analysis for a small number of specimens remains our main concern. Therefore, automated batch analysis characteristic of LC-MS/MS for analyzing massive throughput is not mandatory in this respect. In addition to the shorter turnaround time of TD-ESI/MS, it also supersedes LC-MS/MS in that the former is much less demanding in terms of technical skills. Barely after minimal training, the user-friendliness of TD-ESI/MS platform makes it possible for it to be operated by nurses and physicians working in the emergency departments to identify toxicants in a point-of-care approach and a timely manner. Furthermore, as the thermal desorption unit is much smaller in size than the liquid chromatography unit, the compactness and portability of our TD-ESI/MS platform actualize miniaturization of the analytical tool for its installation in the emergency department with limited space to meet the requirement of efficient bedside applications.

More importantly, the superiority of TD-ESI/MS platform was also documented to the conventional LC-MS/MS platform in its efficiency in identifying toxicants for many other different varieties of acute poisoning in emergency settings, such as ingested pesticides,^58,59^ mis-swallowed medications,^60^ herbal and mushroom toxins,^61,62^ psychoactive drugs,^63^ pesticides causing dermal contamination,^64^ etc. In this study, rapid identification of the adulterants in herbal preparations was demonstrated, we endeavor to add one more invaluable application to the versatility of TD-ESI/MS platform for saving more lives.

A minor limitation of the application of the TD-ESI/MS technique in identifying adulterants in herbal preparations is that thermal desorption might decompose the heat-labile compound of interest altogether. Fortunately, according to our clinical experiences, the majority of the undeclared pharmaceuticals commonly found in herbal preparations to date are heat-stable. Finally, the main challenge we currently confront is the high cost of mass spectrometers and their long-term maintenance in the emergency department from the stakeholders’ point of view. After all, the human lives of the patients who suffer from acute poisoning are priceless!

## 4 Conclusions

The surge in popularity of herbal preparations in Asia has sparked concerns regarding potential adulteration with undisclosed pharmaceuticals, presenting notable public health risks. This adulteration not only jeopardizes the safety and effectiveness of herbal medications but also erodes confidence in natural remedies. To address this issue, a tandem mass spectrometric platform utilizing TD-ESI/MS/MS and probe sampling was devised for swift characterization of improperly adulterated drugs in both herbal preparations and human serum. The operational parameters of this ambient ionization mass spectrometric platform were fine-tuned to enable rapid qualitative analysis within a minimal turnaround time. This development is poised to aid emergency physicians in expeditiously making accurate medical judgments and treatments, significantly enhancing the efficiency of clinical resuscitation. Consequently, it is anticipated to reduce the societal costs associated with subsequent medical care for individuals who have experienced acute poisoning due to adulterants in herbal preparations. Further studies in assessing the capabilities of the TD-ESI/MS platform are necessary to explore its versatility fully. For example, a general multiherbal matrix/multidrug screening procedure requiring no prior knowledge about herbal products, decoctions, or adulterants would make it a more useful analytical tool in emergency care.

## Supporting Information Available

TD-ESI/MS and TD-ESI/MS/MS mass spectra of adulterant standards; LC/MS/MS results of adulterant standards spiked in herbal powders; LC/MS/MS results of adulterant standards spiked in herbal decoctions; and LC/MS/MS results of adulterant standards spiked in human serum (PDF)
