# Supplementary material for: Rapid Characterization of Undeclared Pharmaceuticals in Herbal Preparations by Ambient Ionization Mass Spectrometry for Emergency Care
Source: J Am Soc Mass Spectrom. 2024 Apr 15;35(5):960–71. doi: 10.1021/jasms.4c00016 (PMC11066970; doi:10.1021/jasms.4c00016)
Supplement: Supplementary file 1 — js4c00016_si_001.pdf [file js4c00016_si_001.pdf]

Supporting Information

Full title:

Rapid Characterization of Undeclared Pharmaceuticals in Herbal Preparations by Ambient Ionization Mass Spectrometry for Emergency Care

Short title:

Detection of adulterants in herbal preparations with ambient ionization mass spectrometry

Authors:

Chi-Wei Lee[1,7,8*], Hung Su[2], Yi-Wen Hsu[1], Lin-Zhen Su[1], Yen-Hung Wu[3], Chia-Yi Hou[4], Shu-Yu Shih[5], Jentaie Shiea[2,6,7,8**]

Affiliations:

[1]Institute of Medical Science and Technology, National Sun Yat-Sen University, Kaohsiung 804201, Taiwan

[2]Department of Chemistry, National Sun Yat-Sen University, Kaohsiung 804201, Taiwan

[3]Department of Emergency Medicine, Kaohsiung Medical University Hospital, Kaohsiung Medical University 80756, Taiwan

[4]Department of Clinical Pathology, Chi-Mei Medical Center, Liouying 73659, Tainan, Taiwan

[5]Department of Emergency Medicine, Chi-Mei Medical Center, Liouying 73659, Tainan, Taiwan

[6]Department of Medicinal and Applied Chemistry, Kaohsiung Medical University, Kaohsiung 80756, Taiwan

[7]Rapid Screening Research Center for Toxicology and Biomedicine, National Sun Yat-Sen University, Kaohsiung 804201, Taiwan

[8]Research Center for Environmental Medicine, Kaohsiung Medical University, Kaohsiung 80756, Taiwan

***Corresponding author:**

1. Prof. Chi-Wei Lee, Institute of Medical Science and Technology, National Sun Yat-

Sen University, 70 Lien-Hai Rd, Kaohsiung 804201, Taiwan
E-mail: chiweilee1964@gmail.com
Fax: +886-7-5250151
Telephone: +886-928695731

2. Prof. Jentaie Shiea, Department of Chemistry, National Sun Yat-Sen University, No. 70, Lienhai Rd., Gushan Dist., Kaohsiung 804201, Taiwan
E-mail: jetea@mail.nsysu.edu.tw
Fax: +886-7-5253933
Telephone: +886-910945040

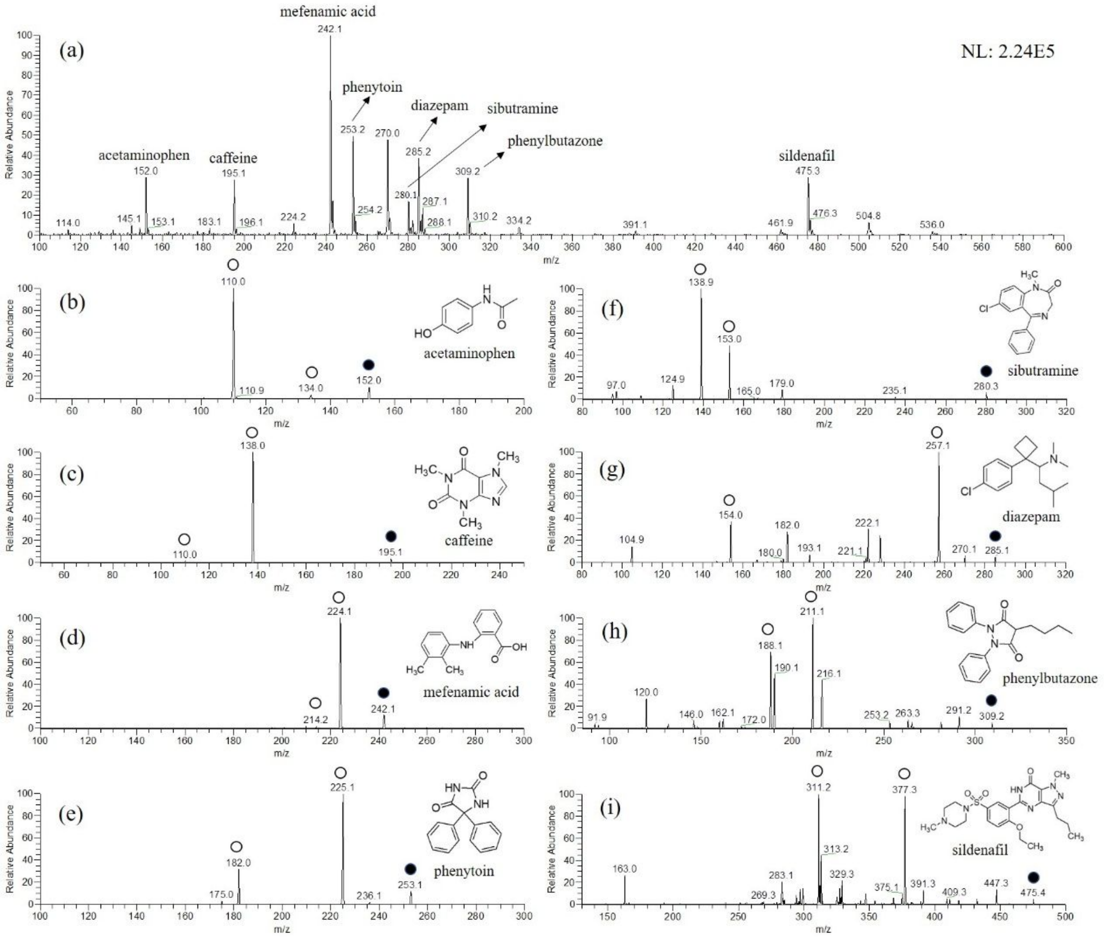

Figure S1. (a) TD-ESI/MS mass spectrum of eight adulterant standards dissolved in pure methanol solution (10 ppm). TD-ESI/MS/MS mass spectra show the precursor and main products ions of each standard: (b) acetaminophen (MH^+, *m/z* 152), (c) caffeine (MH^+, *m/z* 195), (d) mefenamic acid (MH^+, *m/z* 242), (e) phenytoin (MH^+, *m/z* 253), (f) sibutramine (MH^+, *m/z* 280), (g) diazepam (MH^+, *m/z* 285), (h) phenylbutazone (MH^+, *m/z* 309), and (i) sildenafil (MH^+, *m/z* 475). ● Precursor ion, ○ product ion

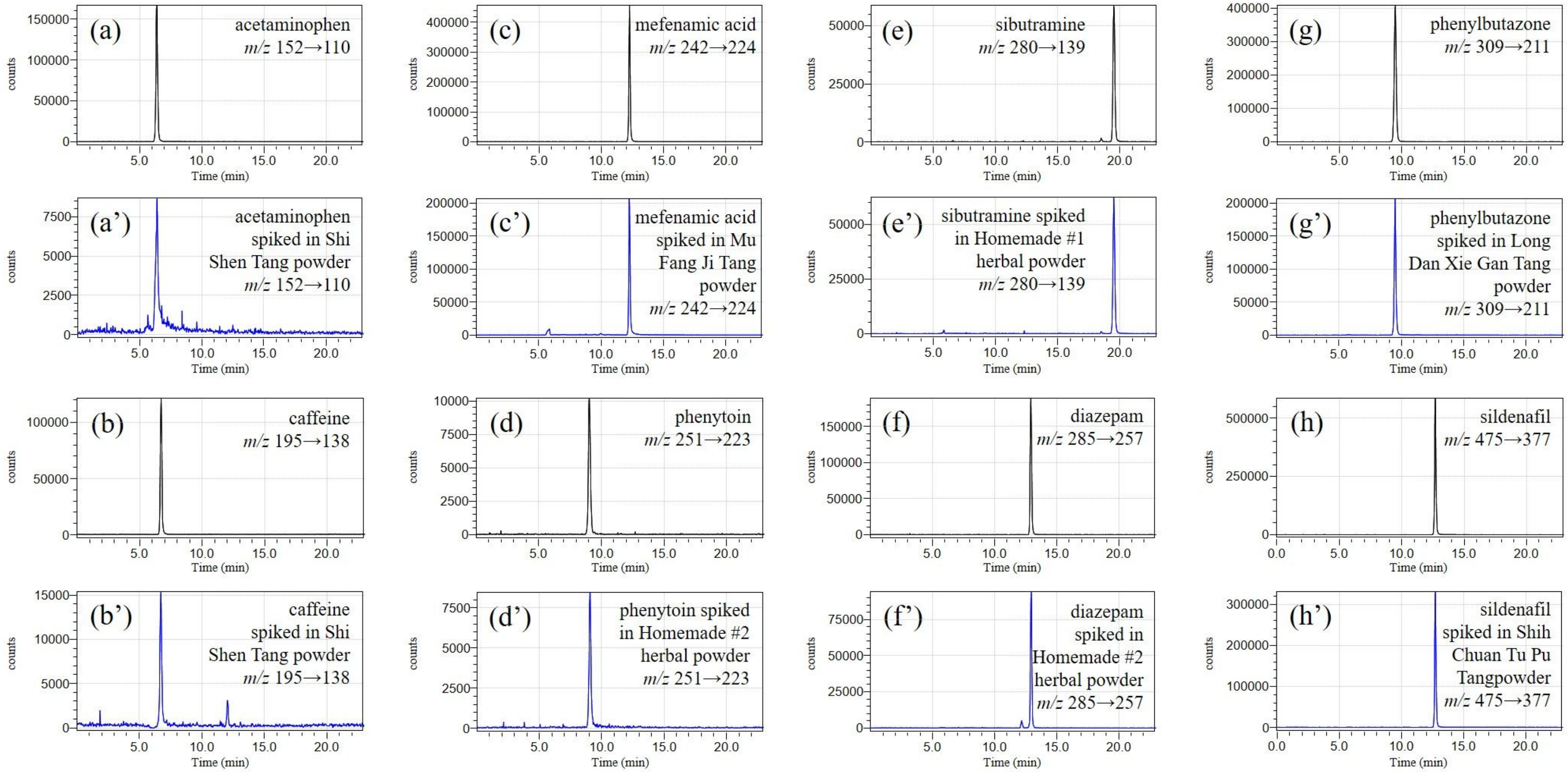

Figure S2. Detection of (a) acetaminophen, (b) caffeine, (c) mefenamic acid, (d) phenytoin, (e) sibutramine, (f) diazepam, (g) phenylbutazone, and (h) sildenafil standards dissolved in methanol (10 ppm) with LC/MS/MS. (a'-h') Detection of the standard spiked in herbal powders by solvent extraction followed by LC/MS/MS.

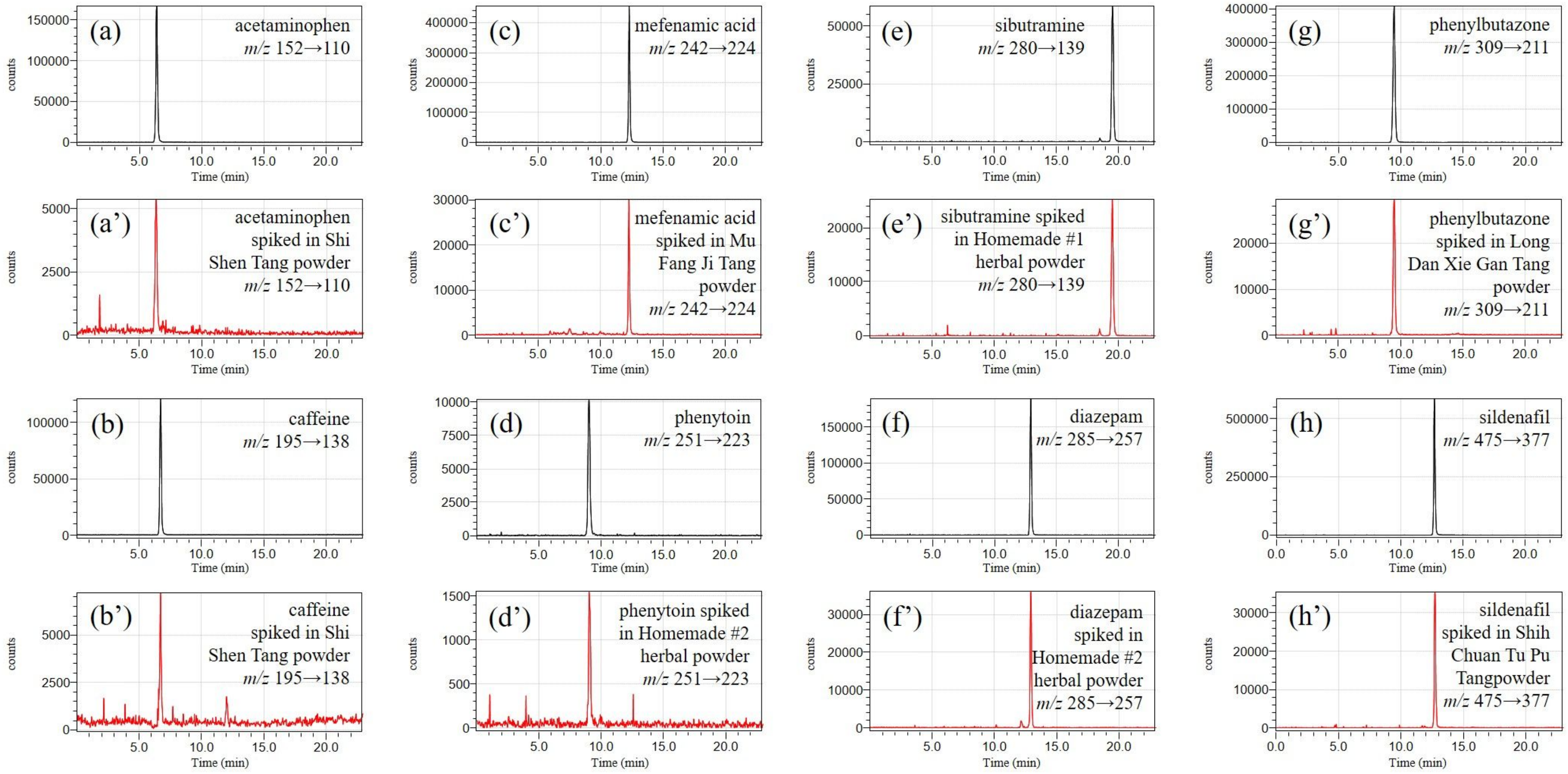

Figure S3. Detection of (a) acetaminophen, (b) caffeine, (c) mefenamic acid, (d) phenytoin, (e) sibutramine, (f) diazepam, (g) phenylbutazone, and (h) sildenafil standards dissolved in methanol (10 ppm) with LC/MS/MS. (a'-h') Detection of the standard spiked in herbal decoctions by solvent extraction followed by LC/MS/MS.

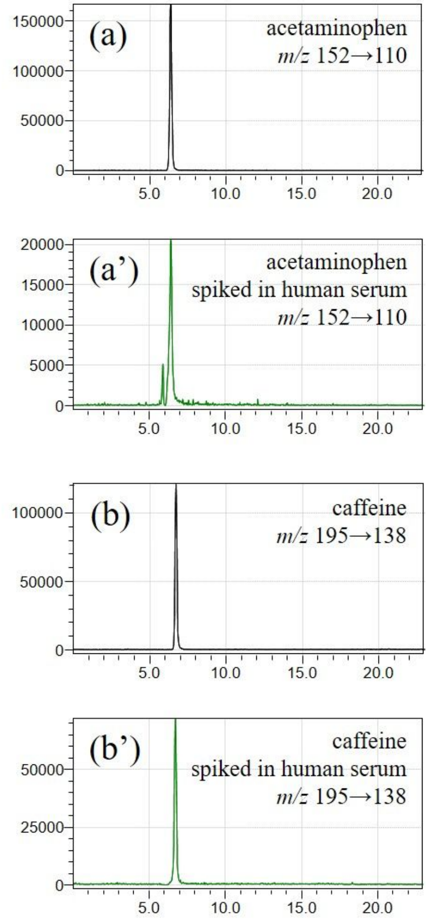

Figure S4. Detection of (a) acetaminophen and (b) caffeine standards dissolved in methanol (10 ppm) with LC/MS/MS. (a',b') Detection of the standard spiked in human serum by solvent extraction followed by LC/MS/MS.
